# Genome-wide survey of B-box proteins in potato (*Solanum tuberosum*)—Identification, characterization and expression patterns during diurnal cycle, etiolation and de-etiolation

**DOI:** 10.1371/journal.pone.0177471

**Published:** 2017-05-26

**Authors:** Urszula Talar, Agnieszka Kiełbowicz-Matuk, Jagoda Czarnecka, Tadeusz Rorat

**Affiliations:** Department of Environmental Stress Biology, Institute of Plant Genetics, Polish Academy of Sciences, Poznań, Poland; Agriculture and Agri-Food Canada, CANADA

## Abstract

Plant B-box domain proteins (BBX) mediate many light-influenced developmental processes including seedling photomorphogenesis, seed germination, shade avoidance and photoperiodic regulation of flowering. Despite the wide range of potential functions, the current knowledge regarding BBX proteins in major crop plants is scarce. In this study, we identify and characterize the *StBBX* gene family in potato, which is composed of 30 members, with regard to structural properties and expression profiles under diurnal cycle, etiolation and de-etiolations. Based on domain organization and phylogenetic relationships, *StBBX* genes have been classified into five groups. Using real-time quantitative PCR, we found that expression of most of them oscillates following a 24-h rhythm; however, large differences in expression profiles were observed between the genes regarding amplitude and position of the maximal and minimal expression levels in the day/night cycle. On the basis of the time-of-day/time-of-night, we distinguished three expression groups specifically expressed during the light and two during the dark phase. In addition, we showed that the expression of several *StBBX* genes is under the control of the circadian clock and that some others are specifically associated with the etiolation and de-etiolation conditions. Thus, we concluded that StBBX proteins are likely key players involved in the complex diurnal and circadian networks regulating plant development as a function of light conditions and day duration.

## Introduction

Plant growth and development are regulated by a wide range of environmental and intrinsic stimuli. The main external stimuli are light and temperature, whereas the fundamental internal stimuli are the circadian clock [1–3], phytohormones [4] and other growth regulatory factors [5]. Plants sense the various spectra of ambient light and convert them into biological signals through the several types of light receptors. Consecutively, photo-activated receptors modulate the activity of downstream signaling components like transcriptional regulators of light- and dark-specific genes. Such signaling pathways finally determine the timing of most developmental transitions during the plant life cycle including germination, the vegetative phase change and the floral transition [6]. On the other hand, the circadian clock synchronizes the physiological and molecular processes to the day/night cycle [1–2].

Zinc finger transcription factors comprise one of the most important family in plants. They play prominent roles in the regulation of plant growth and development. On the basis of structural and functional characteristics of individual members, they are arranged into several distinct subfamilies. Among these, B-box proteins, recently termed BBX [7], have been shown to play essential roles in the light regulatory networks controlling growth and developmental processes such as seedling photomorphogenesis, photoperiodic regulation of flowering, shade avoidance and responses to biotic and abiotic stresses [8–13].

Plant BBX proteins are characterized by the presence in the N-terminus of one single 40-residue B-box domain or two arranged in tandem. B-box domains are classified into two types, known as B-box1 (B1) and B-box2 (B2) depending on their consensus sequence and the distance between the zinc-binding residues [7]. Some members of the BBX family have an additional domain, termed CCT (CONSTANS, CO-like, TIMING OF CAB1: TOC1), composed of 42–43 amino acids located in the C-terminus [7, 8]. While the B-box domain is involved in mediating transcriptional regulation and protein-protein interaction, the CCT domain plays important functions in transcriptional regulation and nuclear protein transport [8]. In addition to these domains, some BBX proteins contain a valine-proline (VP) motif of six amino acids preceding the CCT domain. The BBX family consists of 32 members in Arabidopsis, 30 in rice and 29 in tomato which are classified into five groups based on the presence and number of B-box and CCT domains [7, 13, 14]. In Arabidopsis, groups I and II display two B-box domains and one CCT domain, group III includes one of each domain, group IV possesses two B-box domains and group V only one B-box domain [10].

Transcriptional analysis of several *BBX* genes in Arabidopsis revealed circadian-dependent regulation of expression [15]. Currently, the best characterized plant BBX protein is CONSTANS (CO, AtBBX1) in Arabidopsis. It belongs to group I functions as a transcriptional regulator of the expression of the *Flowering Locus T* (*FT*) gene encoding a “florigen” signal, FT, that triggers flower differentiation under long-day conditions (LD) [16–18]. Conversely, other proteins including AtBBX2 (CO-Like1, COL1) and AtBBX3 (COL2) also belonging to group I do not affect flowering time, but are associated with circadian regulation [19]. Other representatives of groups I and II like AtBBX4 (COL3) and AtBBX7 (COL9) are negative regulators of *CO* and *FT* gene expression, respectively [10, 20, 21]. The current knowledge regarding the function of group-III BBX proteins in Arabidopsis is very limited in plants. One representative, AtBBX16 (COL7), acts as an enhancer of the shade avoidance response in conditions of low red light/far-red light ratio (R/FR) and promotes branching under high R/FR ratio [22]. Group-IV BBXs are mainly involved in regulation of photomorphogenesis. AtBBX20 (BZS1), AtBBX21 (STH2), and AtBBX22 (STH3) proteins specifically promote photomorphogenesis [23–27] whereas AtBBX18 (DBB1a), AtBB19 (DBB1b), AtBBX24 (STO), AtBBX25 (STH) suppress photomorphogenesis [15, 27–29]. These antagonist are associated with BBX abilities to interact with a main photomorphogenesis-promoting factor HY5 [30], with COP1 (CONSTITUTIVE PHOTOMORPHOGENIC 1) and E3 ubiquitin ligase, a dark-dependent repressor of photomorphogenesis mediating degradation of BBX proteins and HY5 factor in the dark [31]. For instance, the interaction of AtBBX21 and AtBBX22 with HY5 enhances its transcriptional activity on target genes [23–25, 32]. In turn, direct interaction of AtBBX24 and AtBBX25 with HY5 suppress this activity due to the formation of inactive heterodimers [27]. Epistatic analyses on BBX proteins and COP1 have shown that BBX4, BBX20, BBX21 and BBX22 repress the COP1 function, whereas BBX24 and BBX25 trigger it [21, 23, 24, 26, 27, 29, 33]. In other respects, the interaction of BBX20, BBX24 and BBX25 proteins with COP1 addresses them to proteasomal degradation [21, 26], whereas BBX21 and BBX22 are recruited by COP1 into nuclear speckles [23, 24]. Note that some members of the group IV are involved in shade avoidance responses by mediating cell elongation [22, 33] but with opposite actions since, BBX19, BBX21 and BBX22 inhibit, and BBX18, BBX24, and BBX25 promote hypocotyl elongation under a low R/FR ratio [33, 34].

In the present study, we characterized the genes encoding the B-box proteins in potato with regard to their structural properties and expression patterns during the day/night phases, etiolation and de-etiolation. We identified 30 genes encoding B-box proteins in the potato genome (*Solanum tuberosum*, ssp. *tuberosum*, cv. Desiree), and classified them into the five structural groups based on the presence of the B-box and CCT domains. According to the time-of-day specificity of their expression during both light and dark phases, we distinguished five distinct types. Further revealed that the expression of eight *StBBX* genes is controlled by the circadian clock and that the transcripts abundance of eleven *StBBX* genes undergoes marked changes during etiolation and de-etiolation conditions.

## Material and methods

### Plant material, growth conditions

*Solanum tuberosum* L., cv. Desiree plants were propagated *in vitro* on solid MS medium at 20/15°C (day/night) under a 150 μmol photon m^−2^ s^−1^ PFD and a 14-h photoperiod, and then planted in soil and grown in a controlled environment chamber under a 250 μmol photon m^−2^ s^−1^ PFD and 14 h light (8:00–22:00) and 10 h dark conditions at 20/15°C, respectively.

For circadian experiments, one set of 2-week-old chamber-grown plants was transferred to continuous light for 2 days under a PFD of 250 μmol photon m^−2^ s^−1^, while a second set of plants was left in the chamber under the same PFD and 14-h photoperiod. Material was collected every 6 hours for 2 days.

For etiolation/de-etiolation experiments, one set of 2-week-old chamber-grown plants was transferred to darkness for 4 days, and then retransferred to 14-h light/10-h dark under a PFD of 250 μmol photon m^−2^ s^−1^. A second set of plants was left in the chamber under the same PFD and a 14-h photoperiod (8:00–22:00). Material from both sets of plants was collected at the following time points: 6:00, 7:55, 8:15, 9:00, 14:00, 20:00 and 14:00 on the following day. Harvested plant material was frozen in liquid nitrogen and stored at -80°C for RNA isolation. Two independent experiments and two independent plant samples for each experiment were used.

### Gene family member identification

The 32 AtBBX protein sequences were used for initial identification of putative potato StBBXs. Preliminary results were found in the Ensembl Plants (http://plants.ensembl.org/) database. For confirmation, we used the National Center for Biotechnology Information (http://www.ncbi.nlm.nih.gov/) database and the PSI-BLAST search tool with algorithm parameters: 500 maximum target sequences by p-value 0.05. To eliminate the probability of omission of any BBX protein in the potato genome, the conserved B-box domain sequence in identified StBBX proteins served as queries to search the database with PSI-BLAST. SMART (http://smart.embl-heidelberg.de/) and Pfam (http://pfam.sanger.ac.uk/), (E-value < 0.05) databases were used to confirm the presence of the B-box and CCT signature, respectively. Full-length cDNA, gene orientation, chromosomal position and protein characteristics were obtained from the Ensembl Plants database. In order to find out additional copies of genes located close to the tandemly arranged *StBBXs*, the coding sequence of identified genes was subjected to BLAST searches using potato pseudomolecule database v 4.04 (E-value < 0.05) [35].

### Gene isolation and sequencing

To determine whether the *StBBX* genes identified by *in silico* analysis are functional, specific primers for each gene were designed within their coding sequence and the corresponding transcripts were cloned and sequenced (S1 Table). Total RNA was prepared from leaves of two-week-old *Solanum tuberosum* plants and the whole flowers of two-month-old plants grown under a 14-h photoperiod using TRIzol Reagent RT (MRC). Equal amounts of total RNA were treated with RNase-free DNase I (Invitrogen). First-strand cDNA was synthesized from 1 μg total RNA using oligo(dT)18 primer and 200 units of Maxima Reverse Transcriptase (Thermo Scientific) at 55°C for 30 min, according to the manufacturer's protocol. The resulting single-strand cDNA was amplified using specific primers for each *BBX* gene (S1 Table). The RT-PCR product was purified from 1% agarose gel using a Gel Extraction Kit (Qiagen) and cloned into the pGEM-T vector (Promega). Two microliters from each ligation were transformed into the XL1-Blue MRF’ *E*. *coli* strain according to the manufacturer’s instructions and selected on LB plates containing 50 μg/ml ampicillin, 2% X-Gal and 100 mM IPTG. Plasmid DNA were isolated from *E*. *coli* with a GeneJET Plasmid Miniprep Kit (Thermo Scientific) and sequenced. The sequencing results were aligned and confirmed with BLAST (blastx).

### Sequence alignment, phylogenetic analysis and *cis*-element identification

The multiple sequence alignment of 30 StBBX proteins was built using MUSCLE in the MEGA 6.06 software with default parameters. The alignment was used as an input to construct the phylogenetic tree with 1000 bootstrap replicates. The alignment of conserved sequences of B-box 1, B-box 2 and CCT domains was generated using ClustalW in MEGA 6.06 and a phylogenetic tree for each domain was constructed using neighbor-joining algorithms with default parameters. The putative StBBX orthologs in tomato and Arabidopsis were identified using the Ensembl Plants (http://plants.ensembl.org/) database. The putative transcription factor binding sites of *StBBX* genes were identified in the genomic sequences within 1000 bp upstream from the start codon using the PlantCARE (http://bioinformatics.psb.ugent.be/webtools/plantcare/html/) database.

### Quantitative real-time PCR analysis

Total RNA was prepared from potato leaves using TRizol Reagent (Invitrogen) according to the manufacturer's protocol. Equal RNA amounts were treated with RNase-free DNaseI. The quantity and quality of RNA were analyzed in 1% agarose gel using an Experion automated electrophoresis station (Bio-Rad). RNA concentration was measured using a NanoDrop photometer (Thermo Scientific). The first DNA strand was prepared using the Maxima Reverse Transcriptase (Thermo Scientific). Real-time PCR was performed using the CFX96 Touch^™^ Real-Time PCR detection system (Bio-Rad, Hercules, USA) and the Maxima SYBR Green/ROX qPCR Master Mix (Thermo Scientific) for 30 *BBX* analyzed genes (S2 Table). Technical duplicates were performed using independent cDNA synthesis reactions. Each assay using gene-specific primers amplified a single product of the correct size with high PCR efficiency (90–110%). The analysis of fluorescence data was conducted using the CFX^™^ Software (Version 3.0, Bio-Rad, Hercules, USA). All qPCR analyses were normalized using the threshold cycle (*C*_1_) values corresponding to the reference genes (*18S* and *EF-1-α*) [36]. The normalized expression of target gene (ΔΔCq) was calculated as the relative quantity of the target gene normalized to the quantities of the two reference genes according to the manufacturer's software, where the denominator of the normalized expression equation is the geometric mean of the relative quantities of the two reference genes for a given sample. The values presented are the means of two technical replicates from two independent biological samples.

## Results

### Identification of the potato *StBBX* gene family

**Fig 1:**
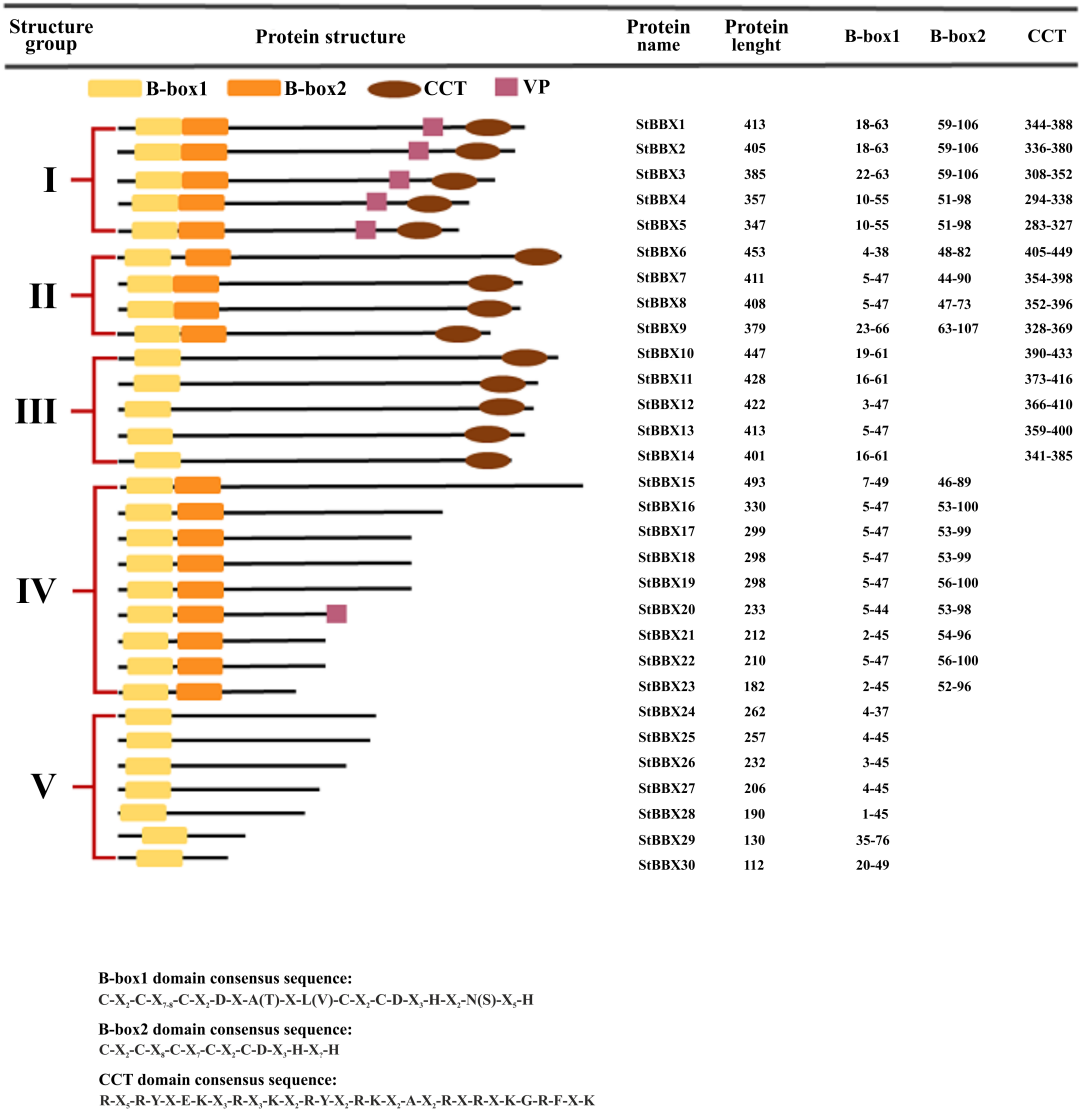
Structural classification of the StBBX family proteins. The name and the length of corresponding StBBX protein and the position of characteristic B-box 1, B-box 2, CCT domains is shown on the right. The location and order of B-box 1, B-box 2 and CCT domains and VP motif within each protein is presented on the diagram.

To identify *B-box* (*BBX*) genes in the potato genome, we searched ENSEMBL PLANTS and NCBI databases using the 32 Arabidopsis BBX sequences as queries. We identified 30 genes encoding BBX proteins with Gene IDs in ENSEMBL PLANTS, and among these only 28 had an accepted LOC number in the NCBI database. The presence of the B-box and CCT domains in identified potato sequences was confirmed using the SMART and Pfam databases, respectively. The consensus sequences of the B-box 1 and B-box 2 zinc finger domains are C-X_2_-C-X_7-8_-C-X_2_-D-X-A(T)-X-L(V)-C-X_2_-C-D-X_3_-H-X_2_-N(S)-X_5_-H and C-X_2_-C-X_8_-C-X_7_-C-X_2_-C-D-X_3_-H-X_?_-H, respectively. The consensus sequence of the CCT domain is R-X_5_-R-Y-X-E-K-X_3_-R-X_3_-K-X_2_-R-Y-X_2_-R-K-X_2_-A-X_2_-R-X-R-X-K-G-R-F-X-K (Fig 1). The identified genes were designated *StBBX*, followed by the Arabic numbers 1–30. The encoded StBBX proteins were classified into five structural groups based on the length, presence and number of characteristic B-box domains and the presence of the CCT domain and VP motif (Fig 1). Group I consists of five representatives, from StBBX1 to StBBX5, containing one double B-box domain, one CCT domain and the VP motif. Group II includes four proteins, from StBBX6 to StBBX9, exhibiting two B-box domains and one CCT domain, whereas proteins belonging to group III, StBBX10-StBBX14, have one domain of each type. Group IV, which includes two B-box domains, is most often represented with nine proteins (StBBX15-StBBX23), while group V is composed of seven members (StBBX24-StBBX30) containing only one B-box domain (Fig 1). Among all the identified *StBBX* genes, only two *StBBX1* (originally designated *StCO*, AM888389) and *StBBX20* (initially termed *SsBBX24*, ABC25454) were previously characterized in potato by Gonzalez-Schain et al. [37] and Kiełbowicz-Matuk et al. [11], respectively.

**Fig 2:**
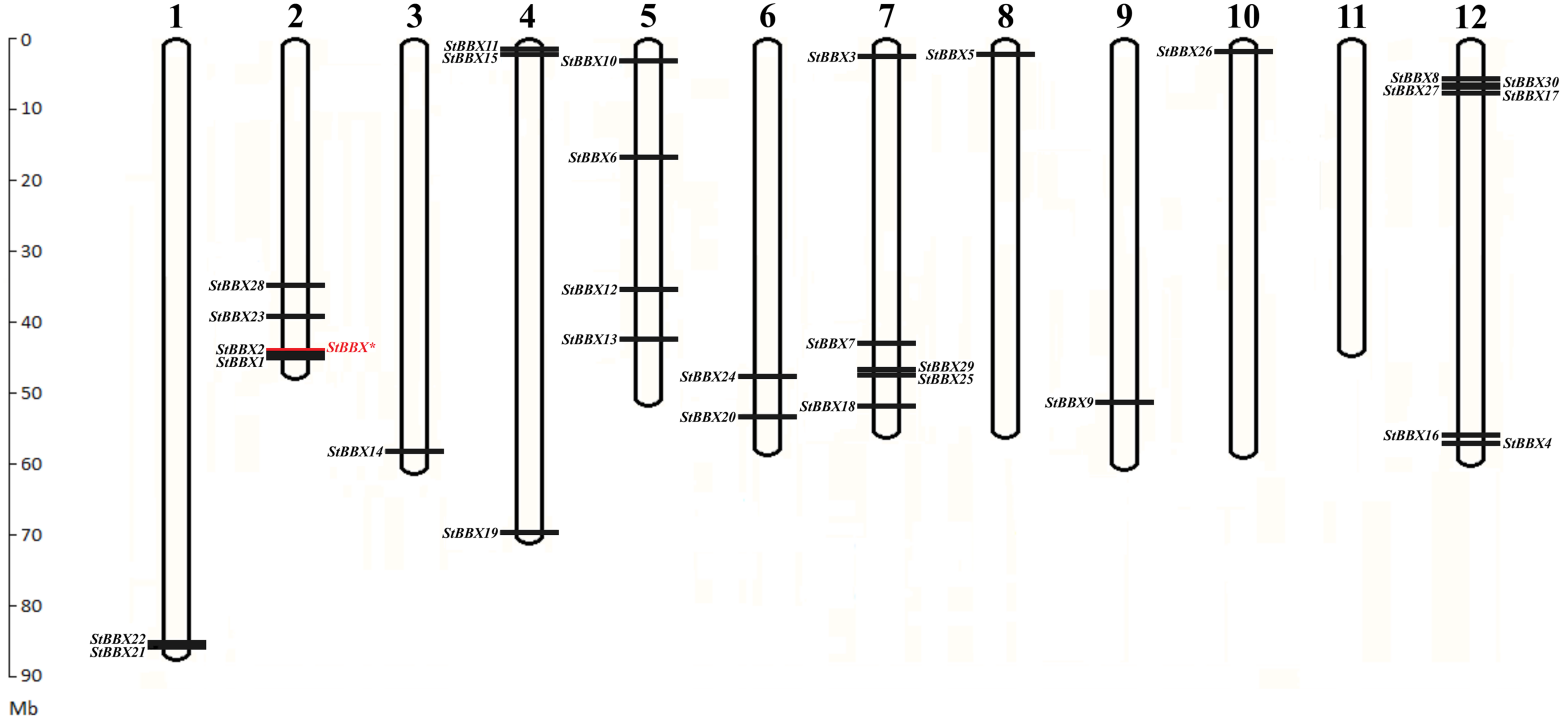
Locations of 30 *BBX* genes on 12 potato chromosomes. The scale on the left is in megabases. Chromosome numbers are indicated at the top of each bar. The gene names on the left and the right side of each chromosome correspond to the approximate locations of each *BBX* gene. An additional copy of StBBX* gene identified using potato pseudomolecule database v 4.04 was marked in red.

When searching through the ENSEMBL PLANTS database, we found that *BBX* genes are widely distributed throughout the potato genome, with the exception of chromosome 11 (Fig 2 and Table 1). Six of these are located on chromosome 12, five on chromosome 7, four on chromosomes 2 and 5, three on chromosome 4, two on chromosomes 1 and 6 and only one is located on chromosomes 3, 8, 9 and 10. In addition, six genes are located in the duplicated segmental regions of chromosomes 1 (*StBBX21*, *StBBX22*), 2 (*StBBX1*, *StBBX2*) and 12 (*StBBX27*, *StBBX30*). The detailed characteristics of *StBBX* genes concerning the gene ID, CDS, orientation, chromosomal position and protein products are shown in Fig 2 and Table 1.

**Table 1:** The *B-box* gene family in *Solanum tuberosum*.

Name	CDS length	Protein length	LOC (NCBI)	GENE ID	Gene position on chromosome	Gene orientation	Accession
***StBBX1***	1242	413	CO CONSTANS	PGSC0003DMG402010056	2:45088023–45092647	forward	KX576511
***StBBX2***	1218	405	LOC102598089	PGSC0003DMG401010056	2:45098374–45101578	forward	KX576512
***StBBX3***	1158	385	LOC102582832	PGSC0003DMG400027475	7:2275710–2277328	reverse	KX576513
***StBBX4***	1074	357	LOC102578495	PGSC0003DMG400029365	12:58153725–58155118	forward	KX576514
***StBBX5***	1044	347	LOC102585080	PGSC0003DMG400026311	8:2052883–2054826	reverse	KX576515
***StBBX6***	1359	453	LOC102603812	PGSC0003DMG400025414	5:16834679–16838305	reverse	KX576516
***StBBX7***	1236	411	LOC102580320	PGSC0003DMG400017411	7:43163087–43172665	forward	KX576517
***StBBX8***	1227	408	ND	PGSC0003DMG400028818	12:6077490–6085194	forward	KX576518
***StBBX9***	1140	379	LOC102582339	PGSC0003DMG400011378	9:52024565–52027713	reverse	KX576519
***StBBX10***	1344	447	LOC102578395	PGSC0003DMG400014566	5:2941260–2943570	reverse	KX576520
***StBBX11***	1287	428	LOC102582864	PGSC0003DMG400007749	4:1419075–1427624	reverse	KX576521
***StBBX12***	1269	422	LOC102594696	PGSC0003DMG400001263	5:42736399–42738599	forward	KX576522
***StBBX13***	1242	413	LOC102603868	PGSC0003DMG400005325	5:35571410–35577321	forward	KX576523
***StBBX14***	1206	401	LOC102595945	PGSC0003DMG400005633	3: 58929268–58930917	reverse	KX576524
***StBBX15***	1482	493	LOC102587007	PGSC0003DMG400005997	4:1835904–1840747	forward	KX576525
***StBBX16***	993	330	LOC102590610	PGSC0003DMG400029426	12:56979384–56982591	forward	KX576526
***StBBX17***	900	299	LOC102593998	PGSC0003DMG400019025	12:7848214–7852389	reverse	KX576527
***StBBX18***	897	298	LOC102587821	PGSC0003DMG400007061	7:52313046–52318239	forward	KX576528
***StBBX19***	897	298	LOC102582815	PGSC0003DMG400003711	4:70449684–70451592	forward	KX576529
***StBBX20***	702	233	LOC102601030	PGSC0003DMG400027017	6:53746105–53749420	forward	KX576530
***StBBX21***	639	212	LOC102578169	PGSC0003DMG400003109	1:86666771–86673688	reverse	KX576531
***StBBX22***	633	210	LOC102593748	PGSC0003DMG400030958	1:86524540–86526577	forward	KX576532
***StBBX23***	549	182	LOC102606359	PGSC0003DMG400003625	2:39712058–39715702	forward	KX576533
***StBBX24***	789	262	LOC102580785	PGSC0003DMG400026515	6:48085593–48086615	reverse	KX576534
***StBBX25***	774	257	LOC102600093	PGSC0003DMG400026181	7:47790598–47791812	forward	KX576535
***StBBX26***	699	232	LOC102587352	PGSC0003DMG400025024	10:1673524–1675193	reverse	KX576536
***StBBX27***	621	206	LOC102585275	PGSC0003DMG400013753	12:6868785–6869802	forward	KX576537
***StBBX28***	573	190	LOC102603949	PGSC0003DMG400022345	2:35131445–35132521	reverse	KX576538
***StBBX29***	393	130	LOC102586344	PGSC0003DMG400026169	7:47281684–47282147	reverse	KX576539
***StBBX30***	339	112	ND	PGSC0003DMG400013178	12:6714370–6714828	reverse	KX576540

To confirm the presence in *S*. *tuberosum* plants of transcripts corresponding to the 30 *in-silico* identified *StBBX* genes, we sought to obtain full-length cDNAs. To this purpose, we isolated total cellular RNA from leaves of two-week-old *S*. *tuberosum* plants grown under a 14-h photoperiod and collected at different time points in the light and dark phases. For cDNA analysis, the samples collected at different time points of the diurnal cycle were combined. RT-PCR analysis revealed the presence of products displaying lengths corresponding to those of genes identified by the *in-silico* approach. In the case of *StBBX12*, the presence of the highest amount of the corresponding transcript was detected in total cellular RNA isolated from the whole flowers of two-month-old *S*. *tuberosum* plants. The RT-PCR products were subcloned into the pGEM-T vector (Promega) and validated by sequencing. Sequencing data revealed that all the 30 isolated *StBBX* genes are identical with the genes annotated in the potato genome.

In order to show whether there are additional copies of *BBX* genes, we searched through the potato pseudomolecule database, and found one additional copy of the gene (with no annotation in the ENSEMBL PLANTS database) located proximally to the *StBBX2* gene in the tandemly arranged *StBBX1* and *StBBX2* on potato chromosome 2 (Fig 2). This additional copy of the gene shares the highest identity with *StBBX2* and *StBBX1* (91.4% and 87.4% identity, respectively). Its putative coding region of 1234 nucleotides does not exhibit a full length of open reading frame, and encodes a truncated protein of 127 amino acid residues that is much shorter compared to the length of StBBX1 and StBBX2 which are comprised of 413 and 405 residues, respectively. The putative truncated protein comprises only the B1 and B2 domains flanked proximally by 20 amino acids and distally by 26 amino acids that allows it to be classified into structure group IV. Analysis of the expression of the putative *BBX* gene shows that the transcript corresponding to the fragment encoding the truncated protein was not detected in leaves of two-week-old *Solanum tuberosum* plants (data not shown). Taking into account the fact that this gene does not exhibit a full reading frame and its expression was not detected, it was not included into the list of the potato *BBX* genes (Table 1).

### Phylogenetic analysis of the StBBX protein family

**Fig 3:**
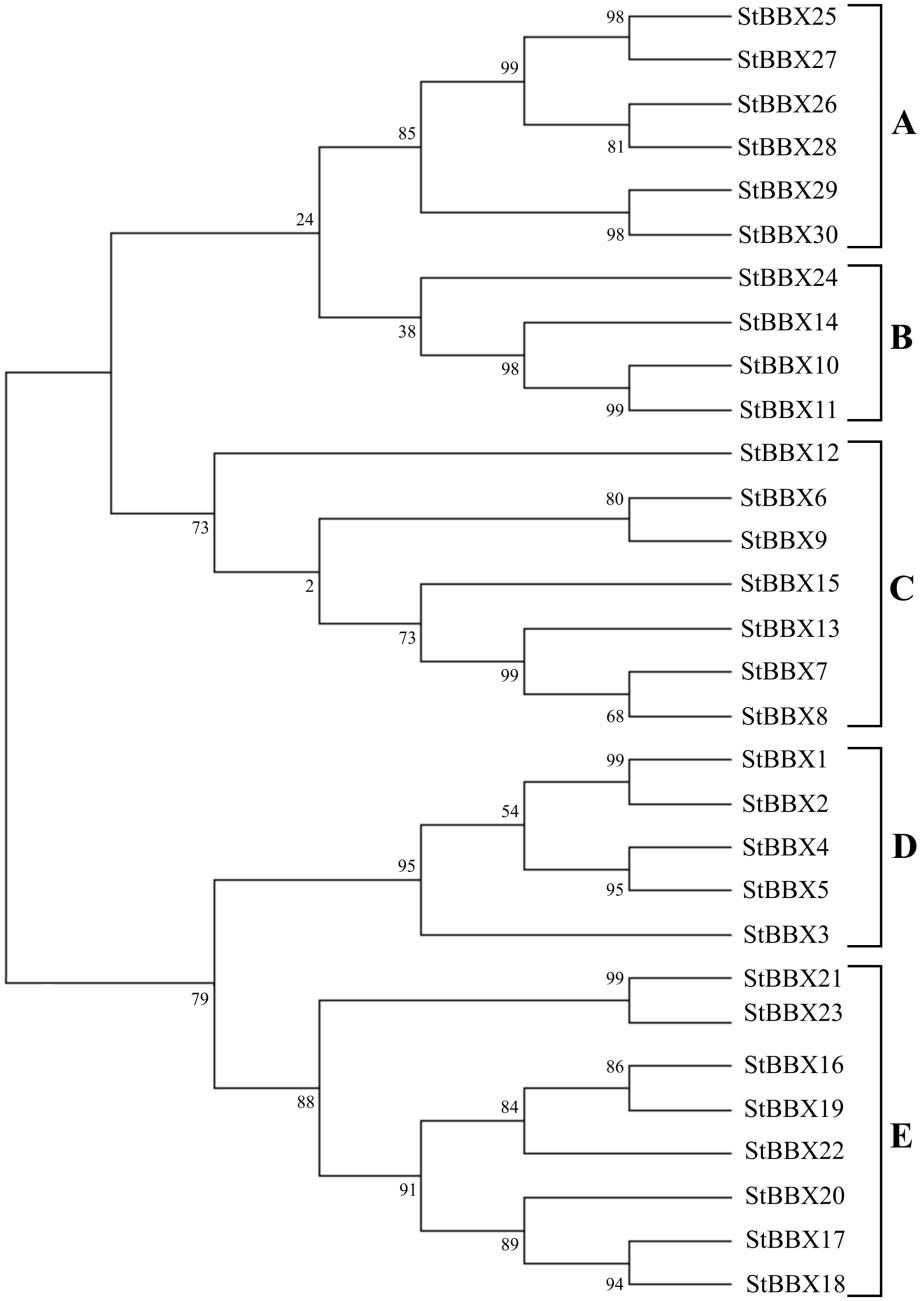
Phylogenetic analysis of the potato B-box family. Full-length proteins of the 30 B-box members were aligned using MUSCLE in MEGA 6.06 software with default parameters [49]. The achieved alignment was used as a input to construct the phylogenetic tree with 1000 bootstrap replicates.

Based on the presence of the B-box and CCT domains and VP motif, the StBBX family proteins were divided into five groups (Fig 1), while the numbering of the proteins within a group was based on their length. To compare the structural classification with the phylogenetic relationships within the StBBX family, we performed a multiple full-length protein sequence alignment and constructed a neighbor-joining tree using MEGA 6.06 software. Based on the phylogenetic analysis, StBBX proteins were classified into five phylogenetic groups (A-E). The results revealed a considerable similarity between the groups defined from the structural features (I-V) and phylogenetic analysis (A-E), with the exception of phylogenetic groups B, C and E (Figs 1 and 3). According to the phylogenetic analysis, the StBBX24 protein classified into structure group V and is located in phylogenetic group B, while the StBBX12 and StBBX13 proteins from structure group III and the StBBX15 protein from structure group IV belong to phylogenetic group C (Fig 3). In addition, we performed other phylogenetic analyzes of the StBBX family based on the multiple alignments of B-box 1, B-box 2 and CCT domains. When aligning B-box 1 domains, we observed a similar phylogenetic relatedness among StBBX proteins compared to that revealed by alignment of full-length sequences (S1 Fig). However, in this classification, the StBBX24 protein localizes to group A (S1A Fig), and not to group B (Fig 3). Moreover, the divisions of the StBBX sequences with regard to the B-box 2 and CCT domains (S1B and S1C Fig, respectively) modify the classification based on the phylogenetic analysis of full-length sequences.

To identify the ortholog of potato BBXs in the tomato and Arabidopsis genome, we performed a BLAST search of the StBBX protein sequences using the Ensembl Plants database and found that orthologs of most B-box proteins in potato were identified in tomato and Arabidopsis (S3 Table).

### Expression of the *StBBX* genes in the diurnal cycle

**Fig 4:**
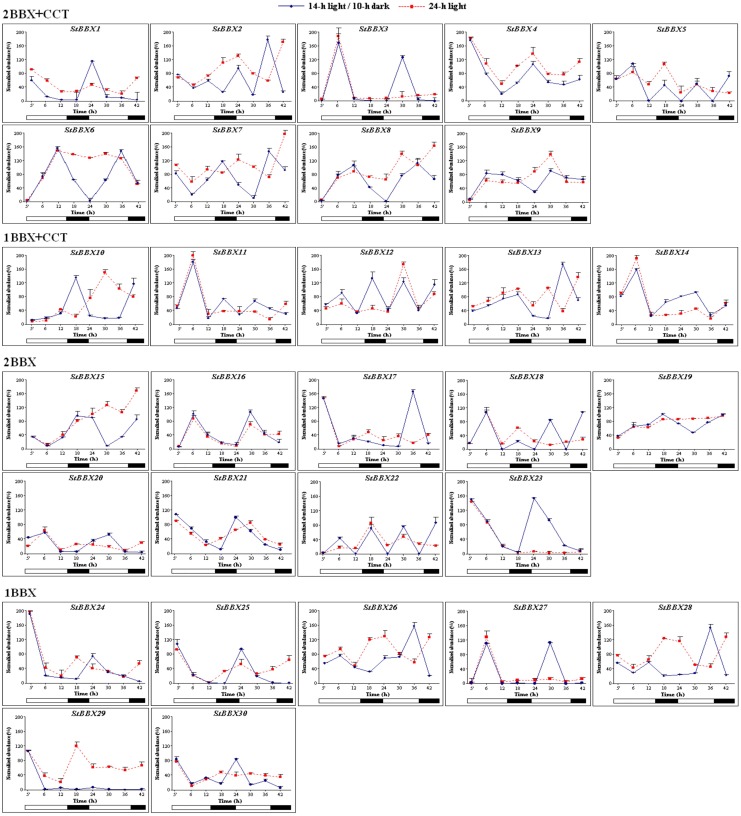
Diurnal and circadian regulation of *StBBX* family genes expression. Analysis of *StBBX* transcript abundances in 2-week-old phytotron-grown *S*. *tuberosum* plants under a 14-h photoperiod (solid blue line) and continuous light (dashed red line) for 42 h during the subjective light and dark phases. Samples were collected at the indicated time points. qRT-PCR was performed as described in Material and Methods. To determine statistical significance between the maximum and minimum levels of the corresponding transcripts oscillation of the *StBBX* genes, we applied the Student's T-test.

**Fig 5:**
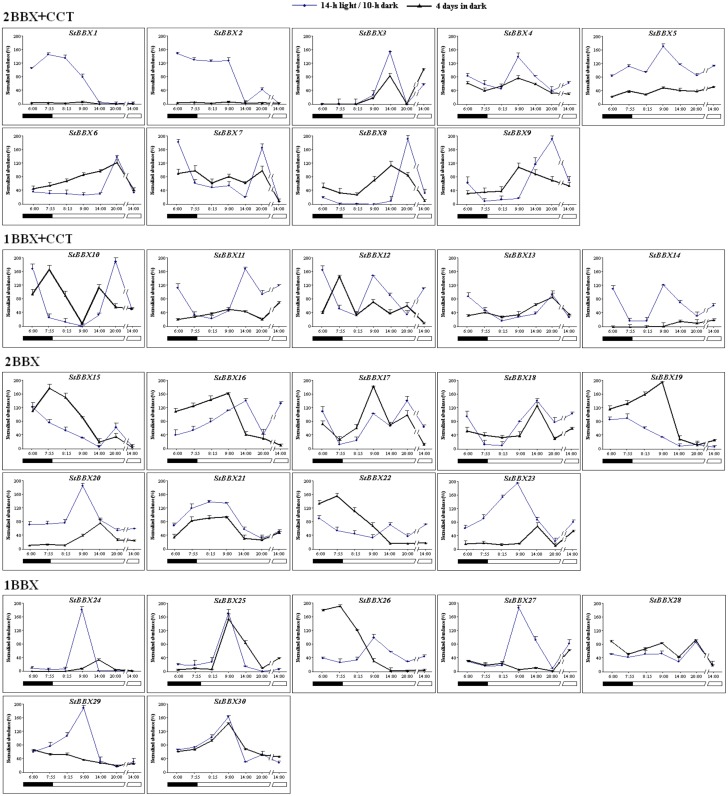
Regulation of *StBBX* family genes expression during etiolation and de-etiolation. Analysis of *StBBX* transcript abundances in 2-week-old phytotron-grown *S*. *tuberosum* plants under a 14-h photoperiod—control plants (solid blue line) and a continuous dark followed by a 14-h photoperiod (black bold line). Samples were collected at the indicated time points. qRT-PCR was performed as described in Material and Methods.

To determine which genes of the *StBBX* family undergo regulation as a function of the light-dark cycle, we first investigated the expression patterns of *StBBX* genes during the diurnal cycle (14h light/10h dark) for 42 h, at 6-hour intervals starting at the beginning of the light phase. As shown in Fig 4, most *StBBX* genes exhibited substantial variations in expression during the diurnal cycle. Interestingly, members belonging to each of the structural groups displayed different expression patterns in the light and dark phases. The diurnal changes in the transcript oscillation of *StBBX* genes between their maxima and minima were significant for most analyzed genes (Fig 4). We observed two main types of expression patterns, the first with maximal expression in the light phase and the second with maximal expression in the dark phase (Table 2). Furthermore, based on the timing of transcript levels we distinguished five different expression groups within these two types. The first and the second groups are light-specific with maximal expression at 6 and 12 hour of the light phase, respectively, while the third and fourth groups are specific for darkness with maximal expression at 4 and 10 hours in the dark, respectively (Table 2). Finally, when compacting the day-time points at the light/dark phase boundary and analyzing the expression of all *StBBX* genes under the light/dark cycle (Fig 5), an additional expression group, with maximal expression at 1 hour of light, was distinguished (Table 2). This expression group includes *StBBX4*, *StBBX21*, *StBBX23*, *StBBX24*, *StBBX25*, *StBBX29* and *StBBX30* genes.

**Table 2:** Classification of *StBBX* genes from *Solanum tuberosum*, cv. Desiree in terms of their expression in diurnal and circadian cycle and etiolation and de-etiolation conditions.

Diurnal-regulated					Circadian-regulated	Etiolation specific	De-etiolation specific
Light specific			Dark specific				
max. expression at 1-h light	max. expression at 6-h light	max. expression at 12-h light	max. expression at 4-h dark	max. expression at 10-h dark			
*StBBX4* *StBBX21* *StBBX23* *StBBX24* *StBBX25* *StBBX29* *StBBX30*	*StBBX3* *StBBX9* *StBBX14* *StBBX16* *StBBX18* *StBBX20* *StBBX26* *StBBX27*	*StBBX6* *StBBX8* *StBBX13* *StBBX17* *StBBX28*	*StBBX10* *StBBX15* *StBBX19*	*StBBX1* *StBBX7*	*StBBX1* *StBBX4* *StBBX9* *StBBX14* *StBBX16* *StBBX20* *StBBX21* *StBBX25*	*StBBX16* *StBBX22* *StBBX26*	*StBBX6* *StBBX8* *StBBX9* *StBBX15* *StBBX17* *StBBX19* *StBBX20* *StBBX23*

For the majority of *StBBX* genes, the changes in the expression profiles observed in the day/night cycle oscillate following a 24-h rhythm (Fig 4). However, large differences were observed between the genes with regard to the amplitude and distribution of maximal and minimal expression levels in the day/night cycle. Collectively, the oscillations were distributed throughout the 24-h cycle (Fig 4). For example, the transcript levels of *StBBX3*, *StBBX16*, *StBBX18* and *StBBX27* increased during the day and reached a maximum in the middle of the light phase, while the expression of others such as *StBBX20*, *StBBX21*, *StBBX23*, *StBBX24*, *StBBX25* and *StBBX30* increased during the night and reached the maximum at the beginning of the light phase (Fig 4). Otherwise, the expression of the *StBBX7*, *StBBX10*, *StBBX15* and *StBBX19* genes was induced in the middle of the light phase, then progressed throughout the subsequent hours, reaching a maximum in the night, and then decreased to the minimum in the first hours of the following light period (Fig 4). Taken together, these data indicate that the phase distribution of the transcript oscillation varied across the day and night; however, twenty *StBBX* genes displayed maximal levels of expression during the light phase.

Next, we investigated the expression of the *StBBX* genes in constant light for 42 hours. Upon continuous light, the expression profile of numerous *StBBX* genes was significantly modified compared to the 14/10-h day/night cycle in terms of the oscillation phase and amplitude. For instance, increased values of minima were noticed for *StBBX2*, *StBBX5*, *StBBX6*, *StBBX9*, *StBBX10*, *StBBX28*, *StBBX29*, and decreased values of the minimum for *StBBX14* and *StBBX25*. An entirely disrupted rhythmicity of the diurnal expression patterns was observed for *StBBX3*, *StBBX7*, *StBBX8*, *StBBX15*, *StBBX18*, *StBBX19*, *StBBX23*, *StBBX26*, *StBBX27*, *StBBX28*, *StBBX29* and an altered phase of cyclic expression by shortening or prolonging the total duration was noticed for *StBBX10*, *StBBX13*, *StBBX21*, *StBBX24*. The expression profiles of a few of the *StBBX* genes including *StBBX1* and *StBBX4* (structure group I), *StBBX9* (structure group II), *StBBX14* (structure group III), *StBBX16*, *StBBX20*, and *StBBX21* (structure group IV), and *StBBX25* (structure group V) were in synchrony under constant light with those observed during the standard day/night cycle (Fig 4), indicating that their diurnal expression is not directly regulated by light, but by the circadian clock (Table 2). Note that expression of *StBBX3*, *StBBX23* and *StBBX27* is totally abolished in the corresponding subsequent light phase upon continuous light (Fig 4), suggesting that diurnal oscillation of their expression requires a dark period.

### Expression of the *StBBX* genes during etiolation and de-etiolation

The expression of *StBBX* genes was analyzed during etiolation and de-etiolation conditions in leaves of two-week-old *S*. *tuberosum* plants. As shown in Fig 5, the transcript levels of *StBBX16*, *StBBX22* and *StBBX26* were substantially higher at the end of the etiolation period compared to those in control plants, and this persisted at a similar level for approximately one hour in the subsequent light phase. Then, the transcript abundances dramatically decreased reaching the levels observed in control plants at the corresponding time-points in the light phase (Fig 5). These data showing increased transcript levels of *StBBX16*, *StBBX22* and *StBBX26* genes in continuous darkness suggest that these genes play physiological functions associated with etiolation processes. In contrast, the transcript abundance of *StBBX6*, *StBBX8*, *StBBX9*, *StBBX15*, *StBBX17*, *StBBX19*, *StBBX20* and *StBBX23* genes was very low by the end of the etiolation period, but dramatically increased in the subsequent hours of re-illumination (Fig 5), indicating that these genes could be involved in de-etiolation processes. Note that expression of the *StBBX6* and *StBBX8* genes in the control plants maintained under 14h light/10h dark conditions was induced after six hours in the light phase, much later compared to the timing of their expression in the control plants shown in Fig 4. Expression of the *StBBX6* and *StBBX8* genes might be very sensitive to any change occurring during the growth of the different sets of plants used for the subsequent experiments. Meanwhile, other *StBBX* genes exhibited widely disparate expression profiles during etiolation and de-etiolation compared to control conditions. Some genes, e.g. *StBBX8*, *StBBX9*, *StBBX16* and *StBBX19*, displayed elevated transcript levels at the end of the fourth day of darkness, followed by a further increase within the following hours in the light, to reach levels similar to those observed in the control plants (Fig 5). Interestingly, the expression level of *StBBX13* was low at the end of the etiolation period, but returned to the level observed in control plants with the onset of light; however, the transcript abundance of others, such as *StBBX27* and *StBBX29*, did not increase in the subsequent light phase (Fig 5). In the case of *StBBX3*, *StBBX21*, *StBBX25*, *StBBX28* and *StBBX30* genes, there was no significant change in their expression profiles during the etiolation and de-etiolation processes (Fig 5).

## Discussion

In the present study, we identified and characterized the potato *StBBX* gene family which is composed of 30 representatives with regard to structural properties and expression profiles under diurnal cycle (day/night phases) and during etiolation and de-etiolation conditions. Based on the presence and the characteristics of B-box and CCT domains and VP motif, *StBBX* genes were classified into five structural groups. On the other hand, based on the time-of-day specificity in expression patterns during light and dark phases, we distinguished several expression groups of *StBBX* genes, three specific for the light phase and two for the dark phase. The changes in the diurnal expression of several *StBBX* genes were shown to be under the control of the circadian clock. Moreover, we also demonstrated that the expression of numerous *StBBX* genes is differentially regulated during etiolation and de-etiolation conditions, indicating that they likely fulfill distinct functions in the diurnal regulation of developmental processes.

Previous phylogenetic analyzes of plant BBX proteins have been based on the genome sequences of *A*. *thaliana* and *Oryza sativa*, followed by comprehensive evolutionary analyzes of families from twelve other plant species by Crocco and Botto [8]. So far, 32, 30 and 29 *BBX* genes have been isolated and characterized in Arabidopsis, rice and tomato, respectively [7, 13, 14]. A uniform Arabidopsis BBX nomenclature was proposed by Khanna et al. [7]. As in Arabidopsis, rice and tomato, potato *StBBX* genes are also widely distributed throughout the chromosomes (Fig 2). The chromosomal localization of *StBBX* genes revealed that some genes were segmentally duplicated on chromosomes 1 (*StBBX21*, *StBBX22*), 2 (*StBBX1*, *StBBX2*) and 7 (*StBBX27*, *StBBX30*), while others were randomly distributed throughout the potato genome. Such a random distribution throughout the potato genome, including segmental duplication events, suggest that distinct mechanisms led to the diversification in the *StBBX* gene family, and that segmental duplication further contributed to an increase in the diversity of *StBBX* genes in potato. The lower number of *BBX* genes in potato genome compared to Arabidopsis may be due to the variable level of genome duplication in the two species. The structural classification of the StBBX proteins was largely consistent with the phylogenetic alignment of the full-length protein sequences, with the exception of four proteins: StBBX12 and StBBX13 belonging to structure group III were classified into phylogenetic group C, despite the lack of one B-box domain (B-box 2); while StBBX15 from structure group IV and StBBX24 from structure group V were classified into phylogenetic groups C and B, respectively, due to the lack of a CCT domain (Fig 3). The genes encoding these StBBX proteins probably lost the B-box 2 domain in evolutionary events, but retained the other common features of their structure group. To better understand the evolutionary origin of the B-box 1, B-box 2 and CCT domains in potato proteins, we performed alignments for each of the conserved domains followed by phylogenetic analyzes. Similar overall classification patterns were observed when B-box 1, B-box 2 and CCT domain sequences were used for the phylogenetic analysis; however, the classification of some StBBX proteins from structural groups II, III and IV has been changed. In addition, phylogenetic analysis of StBBX members enabled the identification of some orthologous genes in other species. For example, *StBBX1* is an orthologous gene of Arabidopsis *BBX1* (*CO*), while *StBBX20* is an orthologous gene of Arabidopsis *BBX24* (*STO*). Further, when searching through the potato pseudomolecule database, we identified an additional copy of the *BBX* gene on chromosome 2 located proximally to the *StBBX2* gene of the tandemly arranged *StBBX1* and *StBBX2*. This finding is in agreement with the data reported for the potato CONSTANS (CO) homologs [38]. These authors also identified three tandemly arranged homologs (*StCOL1-StCOL3*) on potato chromosome 2, and revealed that the *StCOL3* transcript corresponding to the putative potato StBBX* gene (Fig 2) and to tomato *SlCO2* has a deletion in the part encoding the CCT domain, and the transcript is rather undetectable [38].

With the exception of group V, the number of BBX members belonging to each structure group was different in potato and Arabidopsis (Fig 1 and data not shown). However, based on the presence of B-box1, B-box2 and CCT domains in Arabidopsis, rice and potato, we compared the number of proteins belonging to groups I-V and revealed variation between species in terms of the number of representatives in each group. Seven, nine and eleven proteins with two B-box and CCT domains were identified in rice, potato and Arabidopsis, respectively (Fig 1 and data not shown). The number of proteins with one B-box and CCT domain was 10 in rice, 5 in potato and 4 in Arabidopsis, while 10 proteins in rice, 9 in potato and 8 in Arabidopsis contained two B-box domains. The number of proteins with only one B-box domain was 3 in rice, 7 in potato and 7 in Arabidopsis [10, 13]. Based on these data, we suggest that the *BBX* genes from potato, Arabidopsis and rice have a common ancestor. However, differentiations occurred independently on the one hand following first the divergence of dicots and monocots, and on the other following that of *Solanaceae* and *Brassicaceae*.

As reported recently, numerous plant BBX proteins are implicated in multiple light-influenced processes, such as photomorphogenesis, flowering and shade avoidance [37, 39–43]. In Arabidopsis, the CO (AtBBX1) protein is involved in photoperiodic regulation of flowering under long-day (LD) conditions, but has no effect on flowering time under short-day (SD) [16, 18, 40]. Moreover, overexpression of another gene, *COL5* (*BBX6*), causes early flowering in SD conditions in Arabidopsis [41], while overexpression of *COL9 (BBX7)* results in delayed flowering [20]. In rice, *Heading date 1*, the ortholog of Arabidopsis *CO*, shortens the time to heading [42], while *OsCO3* is a floral repressor that regulates flowering under SD conditions [43]. In potato, *StCO* regulates photoperiodic tuberization in a graft-transmissible manner [37]. *Chrysanthemum morifolium* transgenic lines with suppressed expression of *BBX24* flowers earlier than wild-type plants [44]. Given these observations, we determined the characteristics of the expression profiles of all the potato *StBBX* genes in terms of time-of-day and time-of-night specificity. Our results revealed a large diversity in expression patterns during the light and dark phases (Fig 4). The increased expression of numerous *StBBX* genes during the light phase suggests that they are regulated by components of light-signaling pathways, such as phytochromes and downstream regulators of photomorphogenesis, e.g. HY5, LAF1, FAR1, and PHY3 [45]. Other *StBBX* genes are specifically expressed during the dark phase indicating that they could be regulated by dark-specific factors such as *Phytochrome*-*Interacting Factors* (PIFs) that collaborate with phytochromes in the control of gene expression. On the other hand, some genes (*StBBX2*, *StBBX11*, *StBBX13*, *StBBX14*, *StBBX26* and *StBBX28*) display changes in the amplitude of expression patterns during the diurnal cycle at the appropriate time points during 48-h, whereas the overall expression profile is unaltered. This amplitude variation of *BBX* gene expression suggests that post-transcriptional mechanisms contribute to the regulation of these genes. Our data suggest that BBX proteins participate in several developmental processes that are finely regulated as a function of the light and dark phases in the diurnal cycle. The fact that the light- and dark-specific expression groups are represented by members belonging to different structure groups indicates that all BBX types participate in these processes.

We also noticed that the diurnal expression of several *StBBX* genes is controlled by the circadian clock, indicating that these BBX proteins may function as components of circadian clock signaling (Fig 4). This finding is in agreement with the circadian regulation of other *BBX* genes reported for Arabidopsis *BBX2*, *BBX3*, *BBX18*, *BBX19*, *BBX22*, *BBX24*, and *BBX25* [15, 19] and rice [13]. The light- and circadian-dependent expression of the *StBBX* genes is associated with the presence of several light-responsive and circadian-control regulatory elements in their promoter regions (S4 Table). The light-responsive elements may contribute to the regulation of *StBBX* gene expression in the light phase, while the B- and E-box variants of the G-box elements may contribute to the regulation of *StBBX* gene expression in the darkness. In turn, the presence of circadian regulatory elements in the promoters of some *StBBX* genes indicates that they can be controlled by the clock. Current understanding of the mechanisms controlling the expression of plant *BBX* genes is very limited. The best recognized mechanism has been described for the *BBX1* (*CO*, *CONSTANS*) gene in Arabidopsis. *CO* functions as a transcriptional regulator of the expression of the *Flowering Locus T* (*FT*) encoding a florigen hormone FT that triggers flower differentiation under long-day conditions (LD) [17]. To induce the *FT* locus under specific day-length conditions, the timing of daily *CO* transcription and post-translational regulation of CO protein is precisely regulated [46]. As reported by Ito *et* al. [47], expression of the *CO* gene is activated by the FLOWERING BHLH (basic helix–loop–helix) proteins FBH1, FBH2, FBH3, but this is repressed in the morning by the CYCLING DOF FACTOR (CDF) proteins. It was recently revealed that the presence of the CIRC motif in the promoter region of circadian-regulated *StBBX* genes is necessary for the regulation of their expression, and that StZPR1 is a novel nuclear factor that binds the CIRC motif and regulates the circadian expression patterns of genes belonging to the B-box zinc finger family [48].

The light- and dark-specificity of the expression of some *StBBX* genes suggests that they are associated with those processes experienced during etiolation or de-etiolation. Indeed, etiolation-specific (*StBBX16*, *StBBX22*, *StBBX26*) and de-etiolation-specific (*StBBX6*, *StBBX8*, *StBBX9*, *StBBX15*, *StBBX17*, *StBBX19*, *StBBX20* and *StBBX23*) genes have been distinguished (Fig 5 and Table 2). In Arabidopsis, the genes representing the group IV, such as *AtBBX20*, *AtBBX21* and *AtBBX22*, have been shown to promote photomorphogenesis [9, 23–27], whereas *AtBBX18*, *AtBBX19*, *AtBBX24*, *AtBBX25* and *AtBBX32* suppress photomorphogenesis [27–29]. Our data complement those reported for Arabidopsis as they show that expression of the BBX genes classified to group II (*StBBX6*, *StBBX8*, *StBBX9*) is associated with de-etiolation in potato.

In conclusion, we have presented new data highlighting the fact that the expression of most *StBBX* genes is associated with the diurnal cycle; with two groups, this is specifically associated with the light or the dark phase. Furthermore, the expression of most *StBBX* genes oscillates over a period of 24-h, but the amplitude of oscillation and the phase distribution in respect of the light or dark phases are very specific to each gene. Expression of several *StBBX* genes is directly controlled by the circadian clock. Additionally, many *StBBX* genes are associated with etiolation and de-etiolation processes. These data clearly indicate that the *StBBX* genes are involved in diurnal regulatory networks of growth processes regulated by the light and dark, and the circadian clock.

## Supporting information

S1 Fig Phylogenetic analysis of the potato B-box family. The trees shown are based on the alignments of the protein sequences of the B-box 1 domain **(A)**, B-box 2 domain **(B)** and CCT domain **(C)**. The 30 B-box members were aligned using MUSCLE in MEGA 6.06 software with default parameters [49]. The achieved alignment was used as a input to construct the phylogenetic tree with 1000 bootstrap replicates.
(PDF)

S1 Table Primers used for cDNA fragment amplification and sequencing of *StBBX* genes. (PDF)

S2 Table The oligonucleotide primers used for cDNA fragments amplification of *BBX* genes from *S*. *tuberosum* during real-time PCR reactions. (PDF)

S3 Table Potato BBX orthologs in tomato and Arabidopsis. (PDF)

S4 Table *Cis*-regulatory elements involved in diurnal and circadian regulation identified in *StBBX* promoters. (PDF)
