# Supplementary material for: Genome-wide survey of B-box proteins in potato (Solanum tuberosum)—Identification, characterization and expression patterns during diurnal cycle, etiolation and de-etiolation
Source: PLoS One. 2017 May 26;12(5):e0177471. doi: 10.1371/journal.pone.0177471 (PMC5446133; doi:10.1371/journal.pone.0177471)
Supplement: S1 Fig — The trees shown are based on the alignments of the protein sequences of the B-box 1 domain (A), B-box 2 domain (B) and CCT domain (C). The 30 B-box members were aligned using MUSCLE in MEGA 6.06 software with default parameters [49]. The achieved alignment was used as a input to construct the phylogenetic tree with 1000 bootstrap replicates. (PDF) [file pone.0177471.s001.pdf]

S1 Fig.

(A)

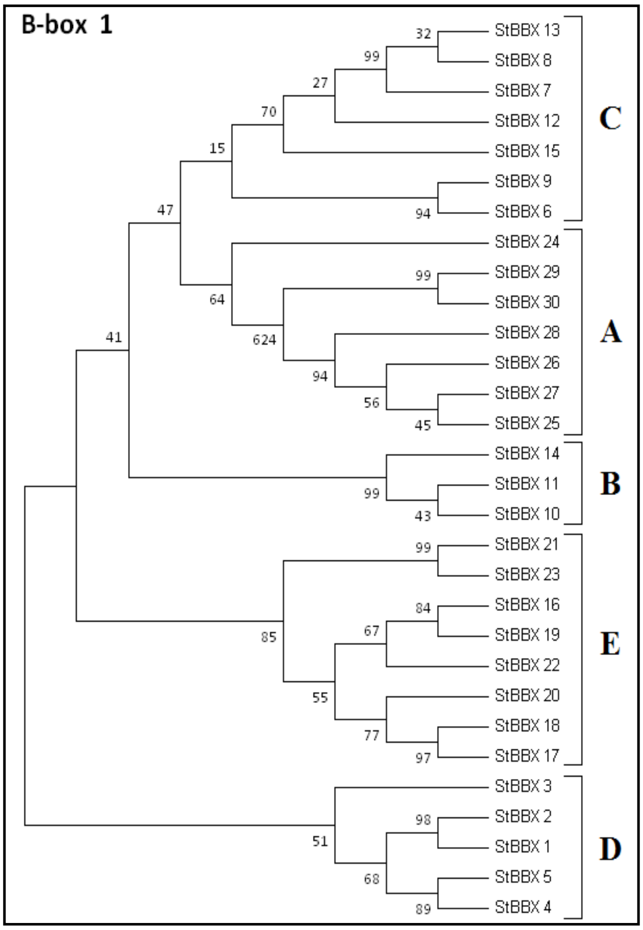

(B)

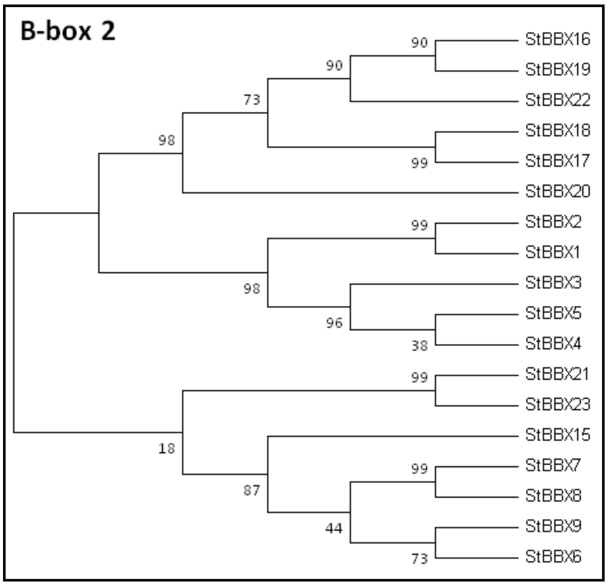

(C)

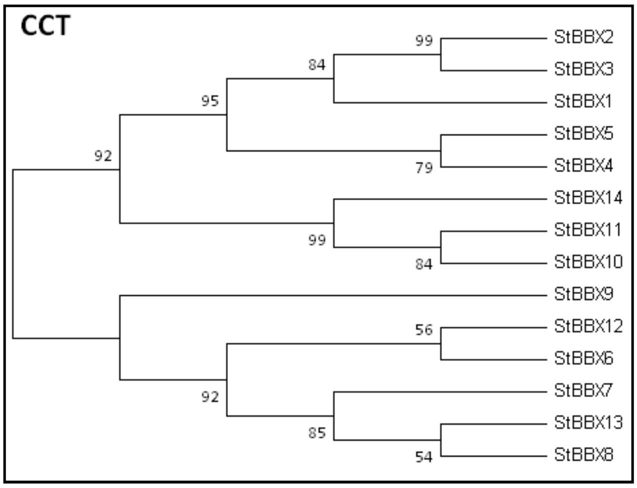

S1 Fig.
Phylogenetic analysis of the potato B-box family.
The trees shown are based on the alignments of the protein sequences of the B-box 1 domain **(A)**, B-box 2 domain **(B)** and CCT domain **(C)**. The 30 B-box members were aligned using MUSCLE in MEGA 6.06 software with default parameters [49]. The achieved alignment was used as a input to construct the phylogenetic tree with 1000 bootstrap replicates.
