# Supplementary material for: Genome-wide survey of B-box proteins in potato (Solanum tuberosum)—Identification, characterization and expression patterns during diurnal cycle, etiolation and de-etiolation
Source: PLoS One. 2017 May 26;12(5):e0177471. doi: 10.1371/journal.pone.0177471 (PMC5446133; doi:10.1371/journal.pone.0177471)
Supplement: S1 Table — (PDF) [file pone.0177471.s002.pdf]

S1 Table. Primers used for cDNA fragment amplification and sequencing of *StBBX* genes.

Gene no.	Forward primer 5'->3'	Reverse primer 5'->3'	Annealing temp.	Cycle no.
StBBX1	TGCTCAACTTCACTACTCCAACT	CTAGGTTTAGGGGCTTGCAGT	62	32
StBBX2	TCAGTATTCGAAGTACAACAGA	ACTAGGTTTAGAGACTTCTAGT	56	36
StBBX3	GCCATCAACATTAGGCTAGA	TGGAATTTTTGGAAATACAACT	59	36
StBBX4	TGATTCTCTCACTTAAACAGA	TCGATTTCAATTTCGGTACG	55	28
StBBX5	TTCACAATAATGGTGGCGGA	GAGTCTCCCATGTCGTTCTC	62	35
StBBX6	CTTTGAATTACAAACATCAAGATT	TATACACACAGAGACAAAAAGA	55	33
StBBX7	AAGTTTCTACCAGAGACCTGA	AACCTCAACCAATTGCCTCT	59	36
StBBX8	AGATCTATACATTGATTCTGGTCT	AAACGGATTGTTTGTTTACCA	58	36
StBBX9	CACCTCTACCAAAACTCCAT	TGCTTATGCAACCTTATTCGG	62	34
StBBX10	AATACACGTATCAGAGGTGG	TGGTCCTTTTAACAAACCTTCC	58	32
StBBX11	AAAGCACGAACACCTAGGCA	AGGAAAAGGGGTTGGTGCAA	67	36
StBBX12	TCAGAGGGATCTGCCCTTTCTA	GTCTTCTGGTATCTGCCCTAGC	64	34
StBBX13	GAACTTGCTAATCTTTGTTTGG	AATCGGTCCTTTGTTTCCTAA	59	34
StBBX14	ACTAGTGGAAGCAAGACGGC	GGCACTGAAAAGGAGGACGA	65	34
StBBX15	GCCCTCTCTAATCGGCATCC	GGCAGGCAAAACTATGAAGACC	66	37
StBBX16	TCCTCTCACTTATAGCAACA	ACATCAGTATAAAATTGGAACGA	57	32
StBBX17	TGTGTGTGAGGTAGCAGAGG	GCCTACTCCTTTTGGAACCG	64	36
StBBX18	TCAATATTAACCATAGATCTTCT	TCACCTATCACAAACATGAG	55	28
StBBX19	TCATCTTCTTCTTCAATTAT	AAATACCCACAGAAATAG	50	37
StBBX20	ATGAAGATCCAGTGTGATGTGT	TTCTGCATCAACCAAGATCTGG	64	28
StBBX21	AATGGCCTGAAGTGGATTCTTG	GCCGGCAACTCCAATTCATAA	66	28
StBBX22	CAGATGAAGCCACACTTTGCC	CGATGTCTTGTGACATTCCTCTGC	68	37
StBBX23	TCTAGGTAATAATATGAGGCTT	ACGAACATTTGCTCATAGAA	55	28
StBBX24	TCCACTATCATAAGTCAAGCTCCA	AATAGAAAATGAAAGAGGGGAGAA	62	34
StBBX25	TGCGTTGAAGTTGTTGCTGT	TCCACGTAATGTTGATTGAAACGA	66	34
StBBX26	AACCCCAAAATAATTCAAATCAC	TCCTACGTACTCATTCCAAACT	59	36
StBBX27	TGTGAATCTGACCACGCGAA	GCCTGTTATAAATCAGCGGCG	68	40
StBBX28	CCCAAATTTGAGATAGAAAAATTA	TCAGATAACAGAATTAGCTTTTGA	59	32
StBBX29	TGTAGTGGAAGAAGAGAGGGTG	GCGTCGTTCTGTAGTCCATC	62	34
StBBX30	TGTGAACTTTGTAAATCAGAAGC	AGAGATACATCAACTTGTCCTTCA	60	32
