# Supplementary material for: Genome-wide survey of B-box proteins in potato (Solanum tuberosum)—Identification, characterization and expression patterns during diurnal cycle, etiolation and de-etiolation
Source: PLoS One. 2017 May 26;12(5):e0177471. doi: 10.1371/journal.pone.0177471 (PMC5446133; doi:10.1371/journal.pone.0177471)
Supplement: S2 Table — (PDF) [file pone.0177471.s003.pdf]

S2 Table. The oligonucleotide primers used for cDNA fragments amplification of *BBX* genes from *S. tuberosum* during real-time PCR reactions.

Name	Primer sequence
StBBX1qRT-Fd	5′ -GCCCCTGCAGCCTTTCTT- 3′
StBBX1qRT-Rev	5′ -GGTGTGAACAGCTGGAGGAC- 3′
StBBX2qRT-Fd	5′ -CTCGACTCGGTTACCCTGCT- 3′
StBBX2qRT-Rev	5′ -CCGTAGGACTCTGGCTTCTC - 3′
StBBX3qPCR-Fd	5′ -CATGGATTCCACCAAACACA- 3′
StBBX3qPCR-Rev	5′ -CACTGAGTGATGGGGTGTTG- 3′
StBBX4qPCR-Fd	5′ -GTGCCGGTGCAGAACAAC- 3′
StBBX4qPCR-Rev	5′ -CTGTGGTCCGGTACAACTCC- 3′
StBBX5qPCR-Fd	5′ -GGATCCGTATGTGGAAATGG- 3′
StBBX5qPCR-Rev	5′ -TATGGTCAGGCACAACTCCA- 3′
StBBX6qPCR-Fd	5′ -TCTGGGGCAGTTGAGGAG- 3′
StBBX6qPCR-Rev	5′ -CATGGGCAAGGGAGCAGT- 3′
StBBX7qPCR-Fd	5′ -ATGCTGTCGAGGGGTCAG- 3′
StBBX7qPCR-Rev	5′ -GAGGAGGCTCCACAGTCTTG- 3′
StBBX8qPCR-Fd	5′ -ACCAAACTGGTGGAGAATGC- 3′
StBBX8qPCR-Rev	5′ -TGGTCGGGAGAGGATCATAG- 3′
StBBX9qPCR-Fd	5′ -TTGGGGTTCCTCCTCTACCT- 3′
StBBX9qPCR-Rev	5′ -TCAACGGACAATTTTGGACA- 3′
StBBX10qPCR-Fd	5′ -ATTAACAGCATGGGCTGACC- 3′
StBBX10qPCR-Rev	5′ -TCTTGGCCTCTTTTCAGCAT- 3′
StBBX11qPCR-Fd	5′ -ACGGCTAGGGCTTGTGAC- 3′
StBBX11qPCR-Rev	5′ -GACTCTGACCCTGACCCTGA- 3′
StBBX12qPCR-Fd	5′ -GCGAGGGCAGTGGTGTAT- 3′
StBBX12qPCR-Rev	5′ -TGAGTCGATGCCCTGTCC- 3′
StBBX13qPCR-Fd	5′ -GCTGTCGAGGGGTCATCA- 3′
StBBX13qPCR-Rev	5′ -GCTCTCTCCCCCAAGGTTAG- 3′
StBBX14qPCR-Fd	5′ -CCTTGTTGGGAGGCGAAG- 3′
StBBX14qPCR-Rev	5′ -CGGGCCAGTAGTCATCAGG- 3′
StBBX15qPCR-Fd	5′ -AGCGAATGGAGCTAAGGTCA- 3′
StBBX15qPCR-Rev	5′ -GGCTACGCAAGCTTAACACC- 3′
StBBX16qPCR-Fd	5′ -ATGTGCAATTGGTGAATGGA- 3′
StBBX16qPCR-Rev	5′ -CTTGAGGGACCCAAATACCA- 3′
StBBX17qPCR-Fd	5′ -GAACCTCTCGGTCCTTCTGC- 3′
StBBX17qPCR-Rev	5′ -GCTGCAGAACCTCCAACC- 3′
StBBX18qPCR-Fd	5′ -TCTTGAGGATCGGGCATTAC- 3′
StBBX18qPCR-Rev	5′ -AGCAGACTCCGGTTCAGAAA- 3′
StBBX19qPCR-Fd	5′ -TTACCTGGATGGCATGTTGA- 3′
StBBX19qPCR-Rev	5′ -GTAGGAGGTGGAGGTGCTTG- 3′
StBBX20qPCR-Fd	5′ -CAAGCTGCAATAAGGAAG- 3′
StBBX20qPCR-Rev	5′ -CTCGACTCATAATCTGGAA- 3′
StBBX21qPCR-Fd	5′ -CGATGGAAGTTCCCTGTGTT- 3′
StBBX21qPCR-Rev	5′ -CAATTTATCCCCCGGAAACT- 3′
StBBX22qPCR-Fd	5′ -TTCAGCAGATGAAGCCACAC- 3′
StBBX22qPCR-Rev	5′ -TCCTGGCAAATGTCACAAAG- 3′
StBBX23qPCR-Fd	5′ -GGCATGTGAGGGTTGGAC- 3′
StBBX23qPCR-Rev	5′ -CCTCTGTAGGCCCAGACTTG- 3′
StBBX24qPCR-Fd	5′ -CGGTGAGGAACGTGTGGT- 3′
StBBX24qPCR-Rev	5′ -GCACTCCCGAGATCTCCTC- 3′
StBBX25qPCR-Fd	5′ -TGTTCCGTGGTCAAATACGA- 3′
StBBX25qPCR-Rev	5′ -CTCGCTTCCACGGAGATG- 3′
StBBX26qPCR-Fd	5′ -GGTGCGTTCGGTGAGTTC- 3′
StBBX26qPCR-Rev	5′ -CTCGCTTCCACGGAGATG- 3′
StBBX27qPCR-Fd	5′ -GAATCTGACCACGCGAATTT- 3′
StBBX27qPCR-Rev	5′ -TCATCGTTCACGCATCTCTC- 3′
StBBX28qPCR-Fd	5′ -GGAGTGGCTCTGGCTCTAAG- 3′
StBBX28qPCR-Rev	5′ -CTTCGTCGTCGTCGTCCT- 3′
StBBX29qPCR-Fd	5′ -AGATCGGTTCACGGAGCTAA- 3′
StBBX29qPCR-Rev	5′ -GGCGTCGTTCTGTAGTCCAT- 3′
StBBX30qPCR-Fd	5′ -TGTGCAATGGAAGAAGAGAAA- 3′
StBBX30qPCR-Rev	5′ -TGCCTTTGAGCCAAGAAATTA- 3′
EF-1-αqRT-Fd	5′ -CTGGTATGGTTAAGATGATTC- 3′
EF-1-αqRT-Rev	5′ -GTCCTTCTTGTCAACATTC- 3′
18SqRT-Fd	5′ -CCAGACATAGTAAGGATTGA- 3′
18SqRT-Rev	5′ -AACGGAATTAACCAGACA- 3′
