# Supplementary material for: Genome-wide survey of B-box proteins in potato (Solanum tuberosum)—Identification, characterization and expression patterns during diurnal cycle, etiolation and de-etiolation
Source: PLoS One. 2017 May 26;12(5):e0177471. doi: 10.1371/journal.pone.0177471 (PMC5446133; doi:10.1371/journal.pone.0177471)
Supplement: S3 Table — (PDF) [file pone.0177471.s004.pdf]

S3 Table. Potato BBX orthologs in tomato and Arabidopsis.

Solanum tuberosum **BBX**	***Solanum lycopersicum*** **BBX**	**% identity***	***Arabidopsis thaliana*** **BBX**	**% identity***
**BBX1**	BBX3	95.91 %	BBX2<br>BBX3	58.03 %<br>61.67 %
**BBX2**	BBX1	90.71 %	BBX3	60.23 %
**BBX3**	BBX6	92.75 %	BBX4<br>BBX6	46.26 %<br>47.32 %
**BBX4**	BBX5	91.90 %	BBX4<br>BBX5	55.44 %<br>46.31 %
**BBX5**	BBX4	92.55 %	BBX4<br>BBX5	52.38 %<br>44.34 %
**BBX6**	BBX12	94.69 %	BBX12<br>BBX13	45.52 %<br>45.03 %
**BBX7**	BBX9	91.39 %	BBX7<br>BBX8	55.65 %<br>54.16 %
**BBX8**	BBX7	95.54 %	BBX7<br>BBX8	58.60 %<br>55.23 %
**BBX9**	BBX11	92.76 %	BBX11	46.08 %
**BBX10**	BBX15	91.76 %	BBX14<br>BBX15	39.66 %<br>40.53 %
**BBX11**	BBX13	77.57 %	BBX14<br>BBX15	26.29 %<br>37.89 %
**BBX12**	BBX10	94.27 %	BBX9<br>BBX10	45.45 %<br>44.78 %
**BBX13**	BBX8	93.41 %	BBX7<br>BBX8	56.72 %<br>53.35 %
**BBX14**	BBX14	77.70 %	BBX14<br>BBX15	33.50 %<br>32.85 %
**BBX15**	BBX27	96.00 %	BBX27	37.08 %
**BBX16**	BBX20	93.62 %	BBX21	43.20 %
**BBX17**	BBX23	92.55 %	BBX22	46.71 %
**BBX18**	BBX22	96.98 %	BBX22	51.10 %
**BBX19**	BBX21	94.65 %	BBX21	45.32 %
**BBX20**	BBX24	97.00 %	BBX24<br>BBX25	62.66 %<br>59.66 %
**BBX21**	BBX19	84.23 %	-	-
**BBX22**	BBX25	95.57 %	BBX 20	47.93 %
**BBX23**	BBX18	97.73 %	BBX18<br>BBX19	59.30 %<br>48.67 %
**BBX24**	-	-	BBX32	31.11 %
**BBX25**	-	-	BBX28<br>BBX29	33.18 %<br>33.02 %
**BBX26**	BBX26	63.46 %	BBX28<br>BBX29	36.77 %<br>32.09 %
**BBX27**	BBX28	84.65 %	BBX28<br>BBX29	32.29 %<br>30.70 %
**BBX28**	BBX29	83.78 %	BBX28<br>BBX29	34.08 %<br>36.28 %
**BBX29**	BBX17	90.00%	-	-
**BBX30**	BBX16	65.14 %	BBX30<br>BBX31	35.04 %<br>32.23 %

*percentage of the orthologous sequence matching the *Solanum tuberosum* sequence
