# Supplementary material for: Genome-wide survey of B-box proteins in potato (Solanum tuberosum)—Identification, characterization and expression patterns during diurnal cycle, etiolation and de-etiolation
Source: PLoS One. 2017 May 26;12(5):e0177471. doi: 10.1371/journal.pone.0177471 (PMC5446133; doi:10.1371/journal.pone.0177471)
Supplement: S4 Table — (PDF) [file pone.0177471.s005.pdf]

S4 Table. *Cis*-regulatory elements involved in diurnal and circadian regulation identified in *StBBX* promoters.

Gene name	Site Name	Organism	Position	Strand	Matrix score	Sequence	Function
BBX1							
	Box 4	*Petroselinum crispum*	657	+	6	ATTAAT	part of a conserved DNA module involved in light responsiveness
	CATT-motif	*Zea mays*	1341	+	6	GCATTC	part of a light responsive element
	G-box	*Zea mays*	835	+	6	CACGTC	*cis*-acting regulatory element involved in light responsiveness
	MNF1	*Zea mays*	1323	+	6.5	GTGCCC(A/T)(A/T)	light responsive element
	Sp1	*Zea mays*	1161	+	5	CC(G/A)CCC	light responsive element
	TCT-motif	*Arabidopsis thaliana*	724	+	6	TCTTAC	part of a light responsive element
	Box II	*Petroselinum hortense*	1240	+	9	TCCACGTGGC	part of a light responsive element
BBX2							
	Box I	*Pisum sativum*	56	+	7	TTTCAAA	light responsive element
	CATT-motif	*Zea mays*	462	+	6	GCATTC	part of a light responsive element
	MNF1	*Zea mays*	927	+	6.5	GTGCCC(A/T)(A/T)	light responsive element
	Sp1	*Zea mays*	614	+	5.5	CC(G/A)CCC	light responsive element
	TCT-motif	*Arabidopsis thaliana*	377	+	6	TCTTAC	part of a light responsive element
BBX3							
	Box 4	*Petroselinum crispum*	21	+	6	ATTAAT	part of a conserved DNA module involved in light responsiveness
	G-Box	*Pisum sativum*	60	+	6	CACGTT	*cis*-acting regulatory element involved in light responsiveness
	G-box	*Solanum tuberosum*	954	+	10	TCACACGTGGC	*cis*-acting regulatory element involved in light responsiveness
	GT1-motif	*Solanum tuberosum*	423	+	10	ATGGTGGTTGG	light responsive element
	Sp1	*Zea mays*	787	+	5.5	CC(G/A)CCC	light responsive element
	circadian	*Lycopersicon esculentum*	568	+	6	CAANNNNATC	*cis*-acting regulatory element involved in circadian control
BBX4							
	LAMP-element	*Pisum sativum*	658	+	8	CTTTATCA	part of a light responsive element
BBX5							
	Box 4	*Petroselinum crispum*	158	+	6	ATTAAT	part of a conserved DNA module involved in light responsiveness
	G-box	*Zea mays*	14	+	6	CACGAC	*cis*-acting regulatory element involved in light responsiveness
	Sp1	*Zea mays*	841	+	5	CC(G/A)CCC	light responsive element
	TCT-motif	*Arabidopsis thaliana*	216	+	6	TCTTAC	part of a light responsive element
	circadian	*Lycopersicon esculentum*	285	+	6	CAANNNNATC	*cis*-acting regulatory element involved in circadian control
BBX6							
	3-AF1 binding site	*Solanum tuberosum*	130	+	10	AAGAGATATTT	light responsive element
	ACE	*Petroselinum crispum*	149	+	9	AAAACGTTTA	*cis*-acting element involved in light responsiveness
	Box 4	*Petroselinum crispum*	356	+	6	ATTAAT	part of a conserved DNA module involved in light responsiveness
	Box I	*Pisum sativum*	543	+	7	TTTCAAA	light responsive element
	G-Box	*Pisum sativum*	410	+	6	CACGTT	*cis*-acting regulatory element involved in light responsiveness
	G-box	*Zea mays*	410	+	6	CACGTT	*cis*-acting regulatory element involved in light responsiveness
	GAG-motif	*Arabidopsis thaliana*	253	+	7	AGAGAGT	part of a light responsive element
	GATA-motif	*Solanum tuberosum*	46	+	9	AAGGATAAGG	part of a light responsive element
	GT1-motif	*Arabidopsis thaliana*	506	+	6	GGTTAA	light responsive element
	circadian	*Lycopersicon esculentum*	856	+	9	CAAAGATATC	*cis*-acting regulatory element involved in circadian control
BBX7							
	Box 4	*Petroselinum crispum*	428	+	6	ATTAAT	part of a conserved DNA module involved in light responsiveness
	Box I	*Pisum sativum*	670	+	7	TTTCAAA	light responsive element
	as-2-box	*Nicotiana tabacum*	508	+	10	GATAatGATG	involved in shoot-specific expression and light responsiveness
BBX8							
	G-box	*Zea mays*	222	+	9	GACATGTGGT	*cis*-acting regulatory element involved in light responsiveness

	GT1-motif	*Arabidopsis thaliana*	949	+	6	GGTTAA	light responsive element
	TCT-motif	*Arabidopsis thaliana*	212	+	6	TCTTAC	part of a light responsive element
	circadian	*Lycopersicon esculentum*	782	+	9	CAAAGATATC	*cis*-acting regulatory element involved in circadian control
BBX9							
	ACE	*Petroselinum crispum*	770	+	9	GACACGTATG	*cis*-acting element involved in light responsiveness
	Box I	*Pisum sativum*	850	+	7	TTTCAAA	light responsive element
	G-Box	*Antirrhinum majus*	772	+	6	CACGTA	*cis*-acting regulatory element involved in light responsiveness
	I-box	*Arabidopsis thaliana*	860	+	9	GATAAGATT	part of a light responsive element
	Sp1	*Zea mays*	724	+	5	CC(G/A)CCC	light responsive element
	circadian	*Lycopersicon esculentum*	554	+	6	CAANNNNATC	*cis*-acting regulatory element involved in circadian control
BBX10							
	AT1-motif	*Solanum tuberosum*	349	+	11	ATTAATTTTACA	part of a light responsive module
	Box 4	*Petroselinum crispum*	349	+	6	ATTAAT	part of a conserved DNA module involved in light responsiveness
	Box I	*Pisum sativum*	272	+	7	TTTCAAA	light responsive element
	G-Box	*Antirrhinum majus*	981	+	6	CACGTA	*cis*-acting regulatory element involved in light responsiveness
	I-box	*Triticum aestivum*	687	+	8	AGATAAGG	part of a light responsive element
	circadian	*Lycopersicon esculentum*	487	+	6	CAANNNNATC	*cis*-acting regulatory element involved in circadian control
	circadian	*Lycopersicon esculentum*	624	+	6	CAANNNNATC	*cis*-acting regulatory element involved in circadian control
BBX11							
	3-AF1 binding site	*Solanum tuberosum*	983	+	10	AAGAGATATTT	light responsive element
	Box 4	*Petroselinum crispum*	78	+	6	ATTAAT	part of a conserved DNA module involved in light responsiveness
	Box I	*Pisum sativum*	963	+	7	TTTCAAA	light responsive element
	GA-motif	*Arabidopsis thaliana*	436	+	8	ATAGATAA	part of a light responsive element
	I-box	*Triticum aestivum*	438	+	8	AGATAAGG	part of a light responsive element
	TCT-motif	*Arabidopsis thaliana*	241	+	6	TCTTAC	part of a light responsive element
	chs-CMA2a	*Petroselinum crispum*	352	+	8	TCACTTGA	part of a light responsive element
BBX12							
	Box 4	*Petroselinum crispum*	466	+	6	ATTAAT	part of a conserved DNA module involved in light responsiveness
BBX13							
	Box 4	*Petroselinum crispum*	305	+	6	ATTAAT	part of a conserved DNA module involved in light responsiveness
	G-box	*Oryza sativa*	905	+	7	GTACGTG	*cis*-acting regulatory element involved in light responsiveness
	G-box	*Daucus carota*	906	+	6	TACGTG	*cis*-acting regulatory element involved in light responsiveness
	LAMP-element	*Pisum sativum*	566	+	8	CTTTATCA	part of a light responsive element
	Sp1	*Zea mays*	211	+	5.5	CC(G/A)CCC	light responsive element
BBX14							
	MRE	*Petroselinum crispum*	164	+	7	AACCTAA	MYB binding site involved in light responsiveness
	TCT-motif	*Arabidopsis thaliana*	256	+	6	TCTTAC	part of a light responsive element
BBX15							
	ACE	*Petroselinum crispum*	264	+	9	CTAACGTATT	*cis*-acting element involved in light responsiveness
	GAG-motif	*Arabidopsis thaliana*	297	+	7	AGAGAGT	part of a light responsive element
	Sp1	*Zea mays*	806	+	5	CC(G/A)CCC	light responsive element
	circadian	*Lycopersicon esculentum*	143	+	6	CAANNNNATC	*cis*-acting regulatory element involved in circadian control
BBX16							
	Box 4	*Petroselinum crispum*	118	+	6	ATTAAT	part of a conserved DNA module involved in light responsiveness
	Box 4	*Petroselinum crispum*	285	+	6	ATTAAT	part of a conserved DNA module involved in light responsiveness
	Box I	*Pisum sativum*	741	+	7	TTTCAAA	light responsive element
	Sp1	*Zea mays*	887	+	5	CC(G/A)CCC	light responsive element
BBX17							
	ACE	*Petroselinum crispum*	688	+	9	GACACGTATG	*cis*-acting element involved in light responsiveness
	Box 4	*Petroselinum crispum*	488	+	6	ATTAAT	part of a conserved DNA module involved in light responsiveness

	G-Box	*Antirrhinum majus*	690	+	6	CACGTA	*cis*-acting regulatory element involved in light responsiveness
	G-box	*Daucus carota*	30	+	6	TACGTG	*cis*-acting regulatory element involved in light responsiveness
	Sp1	*Oryza sativa*	816	+	6	GGGCGG	light responsive element
BBX18							
	G-Box	*Pisum sativum*	465	+	6	CACGTG	*cis*-acting regulatory element involved in light responsiveness
	G-Box	*Pisum sativum*	789	+	6	CACGTT	*cis*-acting regulatory element involved in light responsiveness
	G-box	*Arabidopsis thaliana*	465	+	6	CACGTG	*cis*-acting regulatory element involved in light responsiveness
	G-box	*Zea mays*	789	+	6	CACGTT	*cis*-acting regulatory element involved in light responsiveness
	GA-motif	*Arabidopsis thaliana*	588	+	8	ATAGATAA	part of a light responsive element
	I-box	*Flaveria trinervia*	402	+	10	cCATATCCAAT	part of a light responsive element
	Sp1	*Zea mays*	928	+	5.5	CC(G/A)CCC	light responsive element
	Sp1	*Zea mays*	110	+	5	CC(G/A)CCC	light responsive element
	chs-CMA2b	*Daucus carota*	496	+	11	ATTGCAACTCAA	part of a light responsive element
	circadian	*Lycopersicon esculentum*	731	+	6	CAANNNNATC	*cis*-acting regulatory element involved in circadian control
BBX19							
	3-AF1 binding site	*Solanum tuberosum*	170	+	10	TAAGAGAGGAA	light responsive element
	3-AF1 binding site	*Solanum tuberosum*	192	+	10	AAGAGATATTT	light responsive element
	AT1-motif	*Solanum tuberosum*	975	+	13	AATTATTTTTTATT	part of a light responsive module
	Box I	*Pisum sativum*	754	+	7	TTTCAAA	light responsive element
	G-box	*Brassica oleracea*	807	+	9	TAACACGTAG	*cis*-acting regulatory element involved in light responsiveness
	Sp1	*Zea mays*	249	+	5.5	CC(G/A)CCC	light responsive element
	Sp1	*Zea mays*	263	+	5.5	CC(G/A)CCC	light responsive element
BBX20							
	Box I	*Pisum sativum*	473	+	7	TTTCAAA	light responsive element
	I-box	*Flaveria trinervia*	29	+	10	cCATATCCAAT	part of a light responsive element
	Sp1	*Zea mays*	721	+	5	CC(G/A)CCC	light responsive element
	TCCC-motif	*Spinacia oleracea*	669	+	7	TCTCCCT	part of a light responsive element
	circadian	*Lycopersicon esculentum*	487	+	6	CAANNNNATC	*cis*-acting regulatory element involved in circadian control
BBX21							
	AE-box	*Arabidopsis thaliana*	1199	+	8	AGAAACAA	part of a module for light response
	CATT-motif	*Zea mays*	951	+	6	GCATTC	part of a light responsive element
	Sp1	*Zea mays*	445	+	5	CC(G/A)CCC	light responsive element
	chs-CMA2b	*Daucus carota*	176	+	11	ATTGCAACTCAA	part of a light responsive element
	circadian	*Lycopersicon esculentum*	576	+	6	CAANNNNATC	*cis*-acting regulatory element involved in circadian control
BBX22							
	3-AF1 binding site	*Solanum tuberosum*	839	+	10	AAGAGATATTT	light responsive element
	GT1-motif	*Avena sativa*	291	+	7	GGTTAAT	light responsive element
	MNF1	*Zea mays*	915	+	7	GTGCCC(A/T)(A/T)	light responsive element
	chs-CMA2a	*Petroselinum crispum*	273	+	8	TCACTTGA	part of a light responsive element
	circadian	*Lycopersicon esculentum*	39	+	6	CAANNNNATC	*cis*-acting regulatory element involved in circadian control
BBX23							
	Box I	*Pisum sativum*	156	+	7	TTTCAAA	light responsive element
	GATA-motif	*Arabidopsis thaliana*	332	+	7	GATAGGA	part of a light responsive element
	GT1-motif	*Arabidopsis thaliana*	782	+	6	GGTTAA	light responsive element
	Sp1	*Zea mays*	502	+	5.5	CC(G/A)CCC	light responsive element
BBX24							
	3-AF1 binding site	*Solanum tuberosum*	194	+	10	AAGAGATATTT	light responsive element
	ACE	*Petroselinum crispum*	126	+	9	AAAACGTTTA	*cis*-acting element involved in light responsiveness
	AT1-motif	*Solanum tuberosum*	611	+	11	ATTAATTTTACA	part of a light responsive module
	Box 4	*Petroselinum crispum*	257	+	6	ATTAAT	part of a conserved DNA module involved in light responsiveness
	CATT-motif	*Zea mays*	316	+	6	GCATTC	part of a light responsive element
	GT1-motif	*Arabidopsis*	698	+	6	GGTTAA	light responsive element

		thaliana					
	GT1-motif	*Avena sativa*	744	+	7	GGTTAAT	light responsive element
	L-box	*Petroselinum crispum*	929	+	10	TCTCACCTACC	part of a light responsive element
BBX25							
	Box 4	*Petroselinum crispum*	317	+	6	ATTAAT	part of a conserved DNA module involved in light responsiveness
	G-box	*Solanum tuberosum*	501	+	7	CACATGG	*cis*-acting regulatory element involved in light responsiveness
	G-box	*Zea mays*	661	+	6	CACGTC	*cis*-acting regulatory element involved in light responsiveness
	box II	*Petroselinum hortense*	641	+	9	TCCACGTGGC	part of a light responsive element
	circadian	*Lycopersicon esculentum*	729	+	9	CAAAGATATC	*cis*-acting regulatory element involved in circadian control
BBX26							
	Sp1	*Zea mays*	816	+	5.5	CC(G/A)CCC	light responsive element
	TCCC-motif	*Spinacia oleracea*	564	+	7	TCTCCCT	part of a light responsive element
BBX27							
	G-box	*Zea mays*	708	+	6	CACGTC	*cis*-acting regulatory element involved in light responsiveness
	GT1-motif	*Arabidopsis thaliana*	75	+	6	GGTTAA	light responsive element
	I-box	*Nicotiana plumbaginifolia*	456	+	9	CTCTTATGCT	part of a light responsive element
	TCCC-motif	*Spinacia oleracea*	687	+	7	TCTCCCT	part of a light responsive element
	box II	*Petroselinum hortense*	626	+	9	TCCACGTGGC	part of a light responsive element
	circadian	*Lycopersicon esculentum*	19	+	6	CAANNNNATC	*cis*-acting regulatory element involved in circadian control
	circadian	*Lycopersicon esculentum*	838	+	6	CAANNNNATC	*cis*-acting regulatory element involved in circadian control
	circadian	*Lycopersicon esculentum*	726	+	9	CAAAGATATC	*cis*-acting regulatory element involved in circadian control
BBX28							
	ACE	*Petroselinum crispum*	615	+	9	CTAACGTATT	*cis*-acting element involved in light responsiveness
	Box I	*Pisum sativum*	570	+	7	TTTCAAA	light responsive element
	GATA-motif	*Solanum tuberosum*	820	+	9	AAGGATAAGG	part of a light responsive element
	GT1-motif	*Avena sativa*	392	+	7	GGTTAAT	light responsive element
BBX29							
	ACE	*Petroselinum crispum*	72	+	9	AAAACGTTTA	*cis*-acting element involved in light responsiveness
	CATT-motif	*Zea mays*	972	+	6	GCATTC	part of a light responsive element
	GA-motif	*Arabidopsis thaliana*	927	+	8	ATAGATAA	part of a light responsive element
	Sp1	*Zea mays*	127	+	5	CC(G/A)CCC	light responsive element
	chs-CMA1a	*Daucus carota*	904	+	8	TTACTTAA	part of a light responsive element
	circadian	*Lycopersicon esculentum*	702	+	9	CAAAGATATC	*cis*-acting regulatory element involved in circadian control
BBX30							
	Box I	*Pisum sativum*	974	+	7	TTTCAAA	light responsive element
	GA-motif	*Arabidopsis thaliana*	1163	+	8	ATAGATAA	part of a light responsive element
	Sp1	*Zea mays*	1469	+	5	CC(G/A)CCC	light responsive element
	Sp1	*Oryza sativa*	615	+	6	GGGCGG	light responsive element
	circadian	*Lycopersicon esculentum*	1336	+	6	CAANNNNATC	*cis*-acting regulatory element involved in circadian control
